# miR-665 targets c-MYC and HDAC8 to inhibit murine neuroblastoma cell growth

**DOI:** 10.18632/oncotarget.26046

**Published:** 2018-09-04

**Authors:** Nagindra Prashad

**Affiliations:** ^1^ Genetics in Medicine, Houston, TX 77054, USA

**Keywords:** neuroblastoma, Bt2cAMP, miR-665, c-MYC, HDAC8

## Abstract

Neuroblastoma is a common tumor of the peripheral nervous system in children. Highly aggressive MYC-driven neuroblastoma is defined by increased c-MYC and/or MYCN expression. This study employed a mouse neuroblastoma cell model to assess the role of miR-665 in tumorigenesis. We found that miR-665 suppresses mRNAs, targeting c-MYC and HDAC8, which are involved in neuroblastoma tumorigenesis. N6,2’-O-dibutyryladenosine 3':5'cyclic monophosphate (Bt2cAMP) inhibited neuroblastoma cell growth by inhibiting c-MYC and HDAC8 expression and activating caspase 3. Bt2cAMP also upregulated miR-665, and miR665 transfection mimicked the effects of Bt2cAMP, including reduced c-MYC and HDAC8 expression, increased caspase 3 activation, and reduced neuroblastoma cell growth. As compared to untreated cells, Bt2cAMP increased the number of cells in G1 phase by 50% and the number in G2-M phase by 5%, while the number of cells in S phase was reduced 2.8-fold. Similarly, miR-665 transfection increased the number of cells in G1 phase by 16% and the number in G2M phase by 2%, and decreased the cells in S-phase by 18%. These findings indicate miR-665 suppresses neuroblastoma tumorigenesis by inhibiting c-MYC and HDAC8 expression and suggest miR-665 has potential as an anti-neuroblastoma therapeutic.

## INTRODUCTION

Neuroblastoma is the most frequently diagnosed extracranial solid tumor in children. These tumors account for 15% of childhood deaths from cancer, and clinical outcomes range from spontaneous regression or differentiation into benign ganglioneuromas to aggressive progression and death [1, 2]. Survival in one-year-old children is <30% despite aggressive therapies, including surgery, chemotherapy, radiation therapy, and stem cell transplantation. The MYCN oncogene plays a critical role in neuroblastoma tumorigenesis [3]. However, approximately half of aggressive neuroblastomas lack MYCN amplification [4] and 30% of neuroblastomas with increased MYCN or C-MYC expression show poor survival. One study reported that increased c-MYC and/or MYCN expression defines highly aggressive, MYC- driven neuroblastoma [5]. Similarly, increased HDAC8 levels in neuroblastoma patient tumors correlated with advanced disease [6]. Thus, MYCN, c-MYC, and HDAC8 may each contribute to neuroblastoma tumorigenesis.

MicroRNAs (miRNAs) are small non-coding RNAs that comprise 20–23 nucleotides. miRNAs bind a complementary (or partially complementary) region within the 3’-UTR of a target mRNA to inhibit protein expression through either mRNA degradation or translation inhibition [7]. These molecules are important control switches in cellular development, and are involved in gene expression, proliferation, cell differentiation, and apoptosis [8, 9]. In 2002, Calin, *et al.* [10] reported the first evidence for miRNA involvement in human cancer, suggesting that miRNA-15a and miRNA-16-1 act as tumor suppressors in chronic lymphocytic leukemia. Similarly, oncogenic miR-17-92 overexpression was associated with the human B cell lymphoma [11, 12]. Some anti-cancer drugs inhibit tumor cell proliferation by inducing suppressor miRNA expression [13, 14]. Retinoic acid-induced miR-34a inhibits neuroblastoma cell growth and causes morphological differentiation and apoptosis [15]. Suppressor miR-34a targets MYCN and inhibits neuroblastoma cell proliferation *in vitro* and *in vivo* in mice [16–18]. C-MYC is an oncogenic transcription factor that is activated in many cancers. C-MYC regulates cell proliferation, apoptosis, and cellular metabolism, represses suppressor miRNA expression, and is involved in tumorigenesis in many cancers [19, 20].

Histone deacetylases (HDACs) and acetyltransferases determine histone acetylation status. HDACs are overexpressed in most cancers, leading to histone deacetylation, inhibition of growth suppressive genes, and increased cell proliferation [21]. These epigenetic modifications alter the expression of genes that regulate mRNA and miRNA levels, the cell cycle, and apoptosis [22, 23]. HDAC8 overexpression correlated with advanced neuroblastoma in patient tumor samples, and HDAC8 inhibition reduced cell proliferation and induced neuroblastoma cell differentiation [6]. HDAC inhibitors reduced proliferation and induced apoptosis in neuroblastoma cells *in vitro* and *in vivo* in mice [24, 25]. Several suppressor miRNAs target overexpressed HDACs and inhibit tumor cell growth. miR-449a targets HDAC1 and inhibits prostate cancer cell growth. Similarly, miR-29b targets HDAC4 in multiple myeloma and miR-376a targets HDAC9 in hepatocellular carcinoma [26–28].

We previously reported that N6,2'-O-dibutyryladenosine 3':5'-cyclic monophosphate (Bt2cAMP)-treated murine neuroblastoma cells showed growth inhibition and loss of anchorage independent growth in soft agar. Bt2cAMP treatment also increased cAMP binding protein expression [29, 30]. Our present findings indicated that Bt2cAMP treatment inhibited mouse neuroblastoma cell proliferation, increased caspase 3 activity, and decreased c-MYC and HDAC8 levels. We hypothesized that these effects were mediated by upregulated suppressor miRNAs targeting c-MYC and HDAC8. We found that Bt2cAMP treatment upregulated 18 miRNAs by 1.5–3-fold. Among these, miR-665 most effectively inhibited growth of neuroblastoma cells. Our results demonstrated that miR-665 targets c-MYC and HDAC8 mRNA, miR-665-treatment also increased the percentage of cells in G1 phase and reduced the percentage of cells in S phase of the cell cycle. This is the first report to show that miR-665 is a suppressor miRNA directly targeting the 3’-UTRs of c-MYC and HDAC8 in neuroblastoma.

## RESULTS

### Effects of Bt2cAMP on neuroblastoma cells

**Figure 1:**
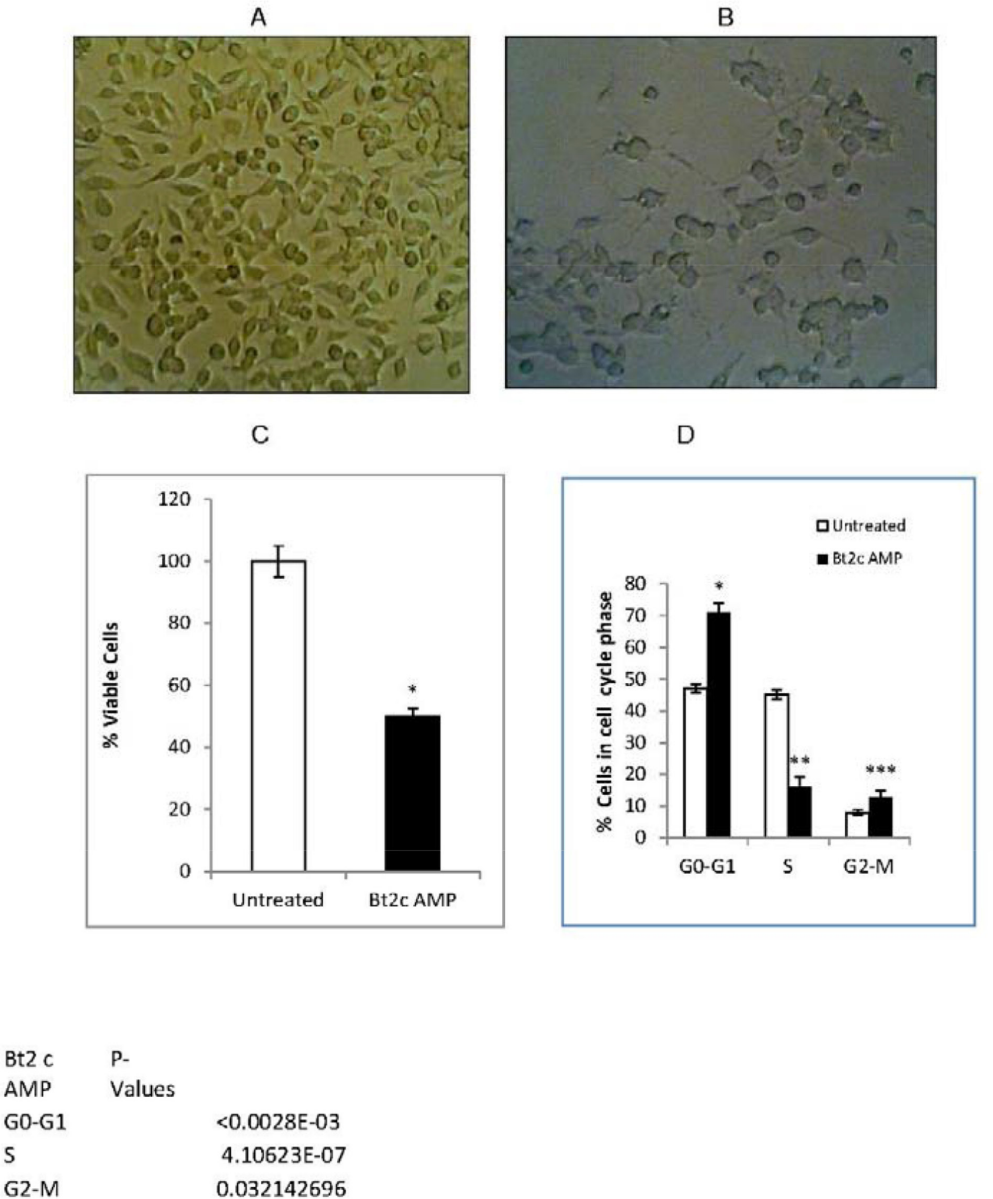
Bt2cAMP induced cell differentiation and inhibited cell proliferation Mouse neuroblastoma cells were grown in monolayers and morphology was observed using a phase contrast microscopy at 100X magnification. Untreated cells **(A)** Cells treated with 1mM Bt2cAMP for 72 h show cell differentiation with neurites **(B)** Bt2cAMP inhibited cell proliferation **(C)** Untreated cells and cells treated with 1mM Bt2cAMP for 72 h were analyzed for cell viability via MTS assay. Viability (%) was compared between untreated (white) and Bt2cAMP-treated cells (black). Data is presented from four independent experiments with two or three biological replicates per experiment. Bars represent SEM. ^*^P<0.02.Untreated cells and cells treated with Bt2cAMP for 48 h were used for cell cycle analysis **(D)** The % of cells in each phase of the cell cycle is shown for untreated (white) and Bt2cAPM-treated cells (black). SEM bars represent the standard deviation of 10 independent experiments. ^*^P=3x10^-6^, ^**^P=4x10^-7^, ^***^P<0.03.

Bt2cAMP-treated neuroblastoma cells became larger, with long neurites, and resembled mature differentiated neuronal cells as compared to spindle- and triangular-shaped untreated cells (Figure 1A–1B). These cells lack MYCN gene amplification, but express c-MYC and are tumorigenic. Bt2cAMP plays a role in microtubule assembly in normal cells, which become flat, elongated, and fibroblastic during this process [31]. Bt2cAMP-treated cells had long neurites (Figure 1B), probably due to microtubule filament formation.

Bt2cAMP treatment inhibited cell proliferation by 50% compared to untreated cells (Figure 1C). Bt2cAMP treatment also increased the number of cells in G1 phase by 50% and those in G2-M phase by 5% compared to untreated cells, while the population of cells in S phase was reduced by 2.8-fold (Figure 1D). Our finding that Bt2cAMP induced cell arrest in G1 phase was similar to the reported effects of Bt2cAMP on S49 lymphoma cell arrest in G1 phase [32]. These results suggest that Bt2cAMP may arrest cells in G1 or G2 phase by inhibiting cell cycle regulators.

c-MYC regulates cell proliferation, apoptosis, and cellular metabolism, represses suppressor miRNA expression, and is involved in tumorigenesis in many cancers, including neuroblastoma [5, 19, 20]. HDAC8 is also involved in the neuroblastoma tumorigenesis [6].

**Figure 2:**
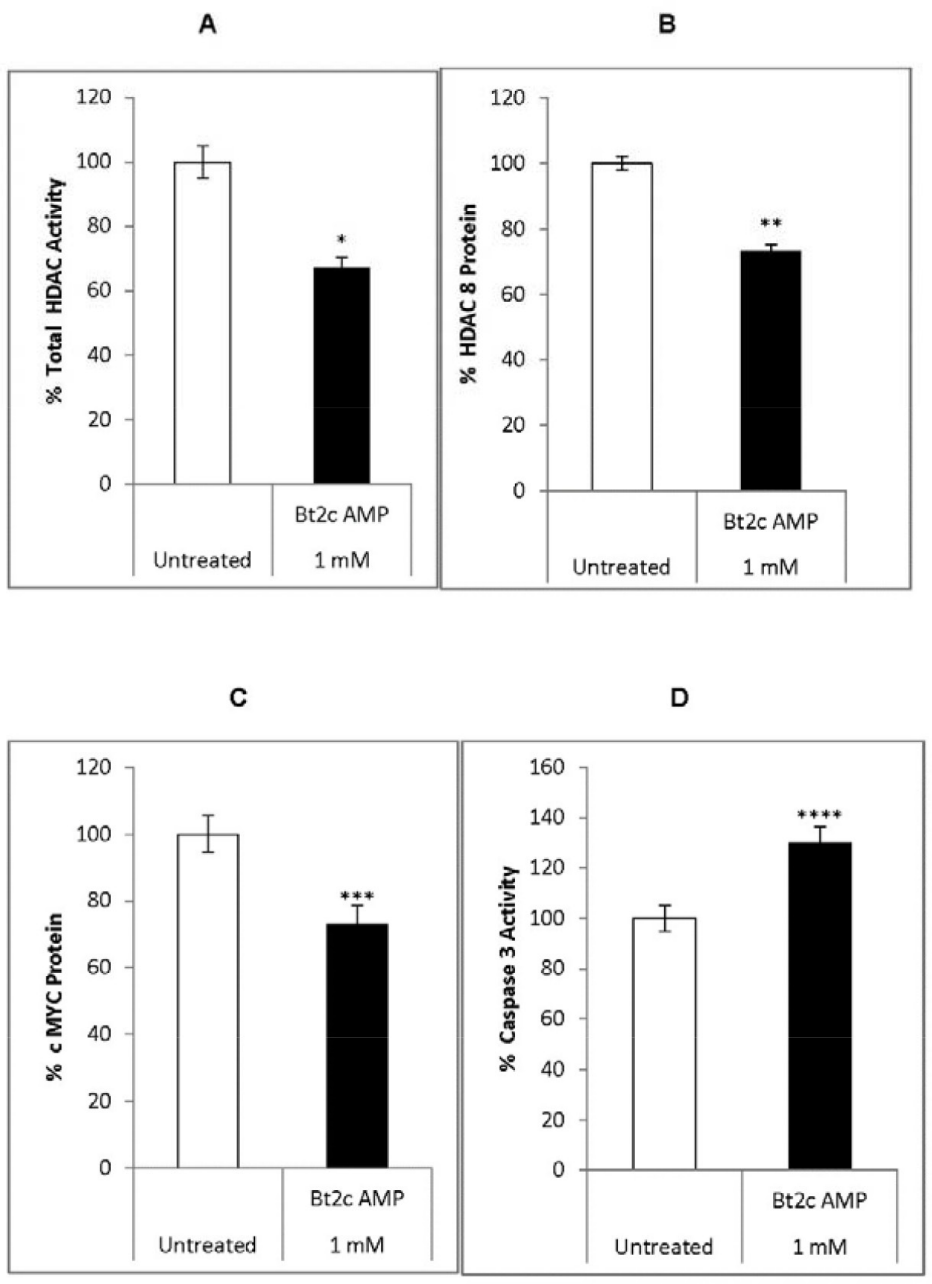
Bt2cAMP effects on gene expression, HDAC activity, and caspase 3 activity Total HDAC activity in untreated (white) and Bt2cAMP-treated cells (1mM Bt2cAMP for 72 h) (black) **(A)** HDAC8 protein levels in untreated (white) and Bt2cAMP-treated cells (black) **(B)** cMYC protein levels in untreated (white) and Bt2cAMP-treated cells (black) **(C)** Caspase 3 activity in untreated (white) and Bt2c AMP-treated cells (black) **(D)** Bars represent the SEM from four independent experiments. ^*^P=7x10^-6^, ^**^P=5x10^-5^, ^***^P=5x10^-5^, ^****^P <0.004.

Therefore, we investigated the effects of Bt2cAMP on c-MYC and HDAC8 expression. Bt2cAMP treatment decreased total pan-HDAC activity by 35% and decreased HDAC8 protein levles by 30% (Figure 2A–2B). Bt2cAMP also decreased c-MYC protein levels by 32% and increased caspase 3 activities by 30% compared to untreated cells (Figure 2C–2D). Sodium butyrate upregulated miR-125-a-5p, inhibited breast cancer cell proliferation, and downregulated both apoptosis and proliferation via miR-22 expression in hepatic cancer cells [33, 34]. Therefore, we speculated that Bt2cAMP might induce suppressor miRNAs in our system.

### Treatment of neuroblastoma cells with Bt2cAMP upregulates miR-655

**Figure 3:**
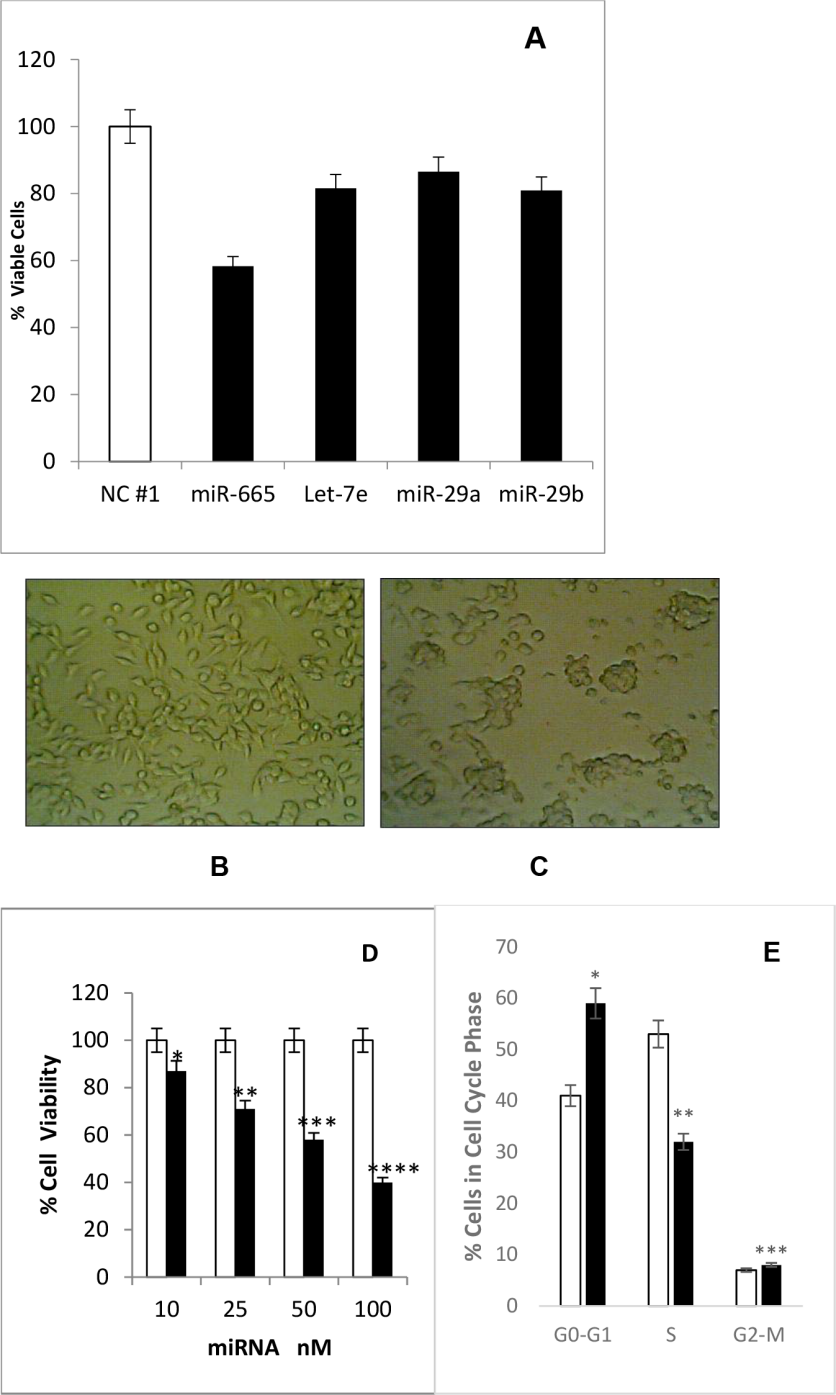
Bt2cAMP effects on miRNAs and cell proliferation **(A)** Four Bt2cAMP-upregulated miRNAs (miR-665, let-7e, miR-29a, and miR-29b) inhibited neuroblastoma cell proliferation. Cell viability was measured via MTS assay. SEM bars represent the standard deviation from three independent experiments with three biological replicates each. ^*^P<0.0005, ^**^P<0.002, ^***^P<0.05. **(B)** Cells transfected with negative control miRNA show a spindle-shaped morphology similar to untreated cells. **(C)** Cells transfected with miR-665 lost their spindle-shape and cells are in clumps. **(D)** miR-665 inhibits cell proliferation in a concentration-dependent manner. Cells were transfected with 10, 25, 50, or 100nM negative control miRNA or miR-665. Cell viability was measured via MTS assay. SEM bars represent the standard deviation from two independent experiments with three biological replicates each. ^*^P<0.04, ^**^P<0.00004, ^***^P<0.0003, ^****^P<0.00001. **(E)** miR-665 transfection increased the number of cells in G1 phase. The % of cells in each phase of the cell cycle is shown for negative control miRNA-transfected (white) and miR-665-transfected cells (black). SEM bars represent the standard deviation from four independent experiments with two biological replicates each. ^*^P<0.001, ^**^P<0.007, ^***^P<0.05.

We hypothesized that Bt2cAMP inhibited neuroblastoma cell growth by inducing suppressor miRNAs targeting c-MYC and HDAC8. Therefore, miRNA levels were compared between 1mM Bt2cAMP-treated mouse neuroblastoma cells and untreated cells. 21 miRNAs were differentially expressed in Bt2cAMP-treated cells. 18 of these miRNAs were up-regulated by 1.5–3.0-fold, and three were down-regulated by 2–4-fold compared to untreated cells (Table 1). Up-regulated miRNAs were screened for their effects on neuroblastoma cell growth. Cells were transfected for 72 h with 50 or 100mM negative control miRNA or up-regulated miRNA mimic. Four of these miRNAs (miR-665, let7e, miR-29b, and miR-29c) inhibited cell growth compared to negative control miRNA-treated cells, with miR665 the most effective (Figure 3A). We hypothesized that miR-665 may be a tumor suppressor miRNA that acts on oncogenic targets to inhibit cell growth. We then identified the miR-665 targets responsible for neuroblastoma tumorigenesis.

**Table 1:** miRNA differential expression in Bt2cAMP-treated neuroblastoma cells

Upregulated miRNA	Increased by 2-fold T/C	p-value
miR-665	2-fold	1.35E-06
Let-7e	2-fold	5.00E-05
miR-29a	2-fold	2.20E-05
miR-29b	2-fold	3.50E-05
miR-212	2-fold	9.70E-05
miR-378	2-fold	2.60E-05
miR-132	2-fold	2.70E-05
miR-197	2-fold	1.50E-06
miR-714	3.4-fold	3.30E-05
miR-128		
miR-126-3p		
miR-712		
miR-152		
miR-33		
miR-298		
miR-328		
miR-690		
miR-339-5p		
miR-106b^*^		
miR-470		
miR-29^*^		
miR-878-5p		
miR-880		

Down Regulated miRNA	Decrease by	p-value
miR-210	4-fold	4.60E-06
miR-218^*^-2	2-fold	5.80E-05
miR-149	1.5-fold	6.40E-05

RNA was extracted from untreated cells and cells treated with 1mM Bt2cAMP for 72 h. 18 miRNAs were upregulated 1.5–3-fold and three were downregulated 2–4-fold (P<0.001).

### Transfection with miR-665 arrests neuroblastoma cells in G1 phase

We analyzed the effects of miR-665 on cell morphology and cell cycle progression in neuroblastoma cells. miR-665 transfected cells exhibited morphological changes, losing their normal spindle shape and growing in cellular clumps without process (Figure 3B–3C). Negative control miRNA-transfected cells resembled untreated cells. miR-665 inhibited neuroblastoma cell growth in a concentration-dependent manner by 13% (at 10nM) to 60% (at 100nM) (Figure 3D). Cell cycle analysis was performed using cells transfected with 100nM negative control miRNA or 100nM miR-665 mimic for 72 h. miR-665 transfection increased the number of cells in G1 phase by 16% and those in G2-M phase by 2%, and decreased cells in S-phase by 18% compared to negative control miRNA (Figure 3E). c-MYC indirectly regulates the cell cycle transition from G1 to S phase and activates cell growth-related genes, including E2F genes. E2F1 is involved in DNA replication at G1/S phase of cell cycle [35]. c-MYC downregulation in miR665-treated cells may reduce E2F1 activation, leading to an accumulation of cells in G1 phase.

### miR-665 targets HDAC8 and c-MYC

**Figure 4:**
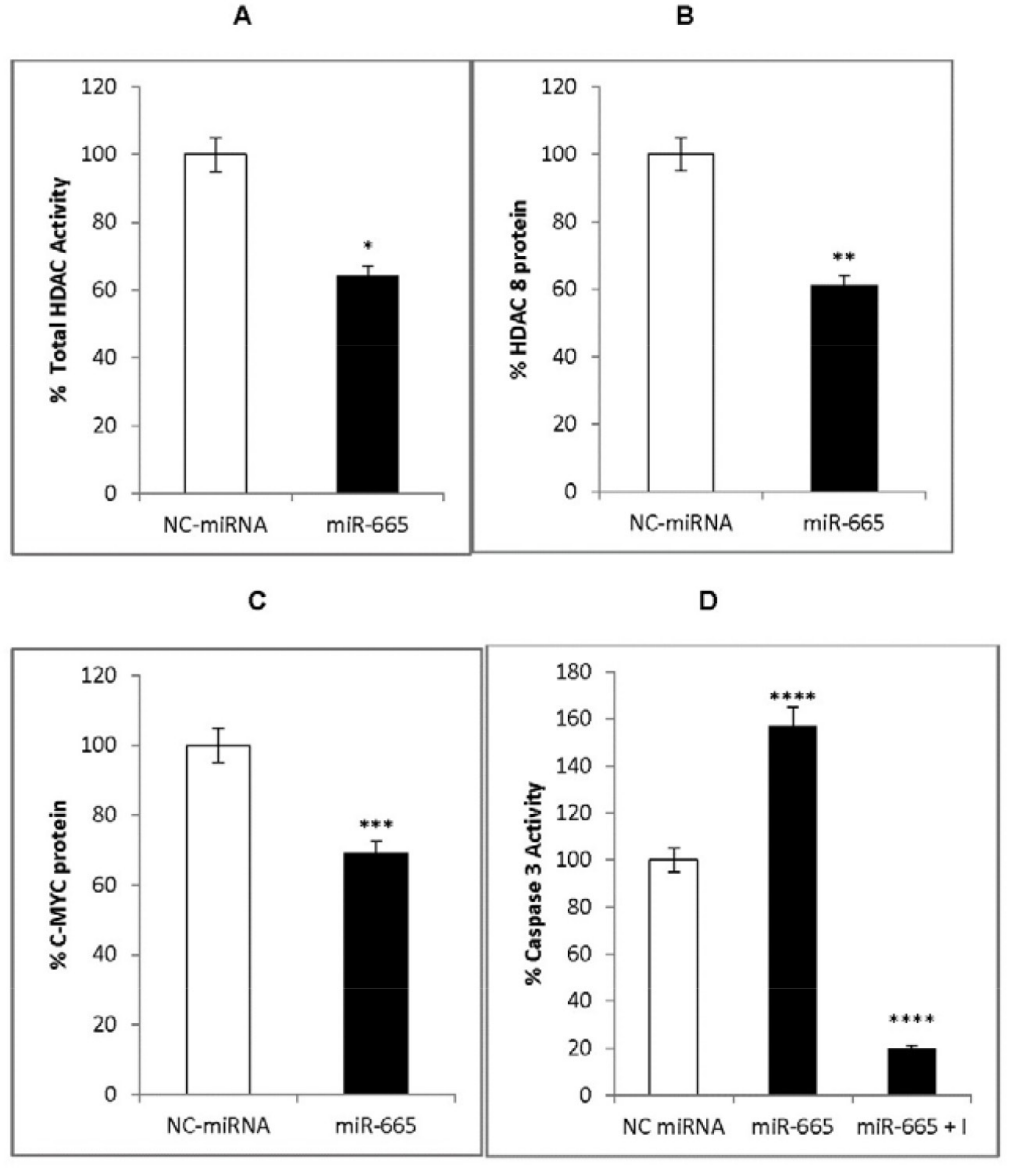
miR-665 effects on HDAC8 and c-MYC expression Total HDAC activity decreased in miR-665-transfected cells (black) compared to negative control miRNA-transfected cells (white) **(A)**, HDAC8 was down regulated in miR-665-transfected cells (black) compared to negative control miRNA-transfected cells (white) **(B)**, c-MYC was down regulated in miR-665 transfected cells (black) compared to negative control miRNA-transfected cells (white) **(C)**, Caspase 3 activity increased by 1.5-fold in miR-665-transfected cells (black) compared to negative control miRNA-665 transfected cells (white) and the inhibitor, Ac-DEVD-CHO, decreased caspase 3 activity (black) **(D).** SEM bars represent the standard deviation from four independent experiments with two biological replicates each. ^*^P<0.008, ^**^P<0.02, ^***^P=3x10^-6^, ^****^P<0.03.

To determine the mechanism by which miR-665 inhibited neuroblastoma cell proliferation, we investigated the effects of miR-665 on HDAC8 and c-MYC expression. Total HDAC activity was measured in the presence of an acetylated HDAC substrate, Ac-Lys (Ac)-p NA. The deacetylated end product was measured colorimetrically. HDAC8 and c-MYC proteins were measured via ELISA. Total HDAC activity, and both HDAC8 and c-MYC levels were decreased by 40% in miR-665-transfected cells compared to cells treated with negative control miRNA (Figure 4A–4C).

### miR-665 activates caspase 3

HDAC inhibitors induce caspase 3-dependent apoptosis in neuronal cells [36]. The tumor suppressor, miR-34a, increases caspase 3 activation, leading to caspase-dependent apoptosis in neuroblastoma cells [15, 17]. Since miR-655 inhibits HDAC8, it would also be expected to induce caspase-dependent apoptosis. Caspase 3 activation was measured through hydrolysis of the peptide substrate attached to p-nitroanilid, Ac-DEVD-pNA. miR-665 increased caspase 3 activity by 1.5-fold compared to cells transfected with negative control miRNA (Figure 4D). The specificity of caspase 3 activity was determined by adding the caspase 3 inhibitor, Ac-DEVDCHO, to reactions before the substrate. The inhibitor reduced miR-665-induced caspase 3 activity by 90% (Figure 4D), indicating specific miR-665-induced activation of caspase 3, which may be involved in caspase 3-dependent apoptosis.

### miR-665 levels following transfection

**Figure 5:**
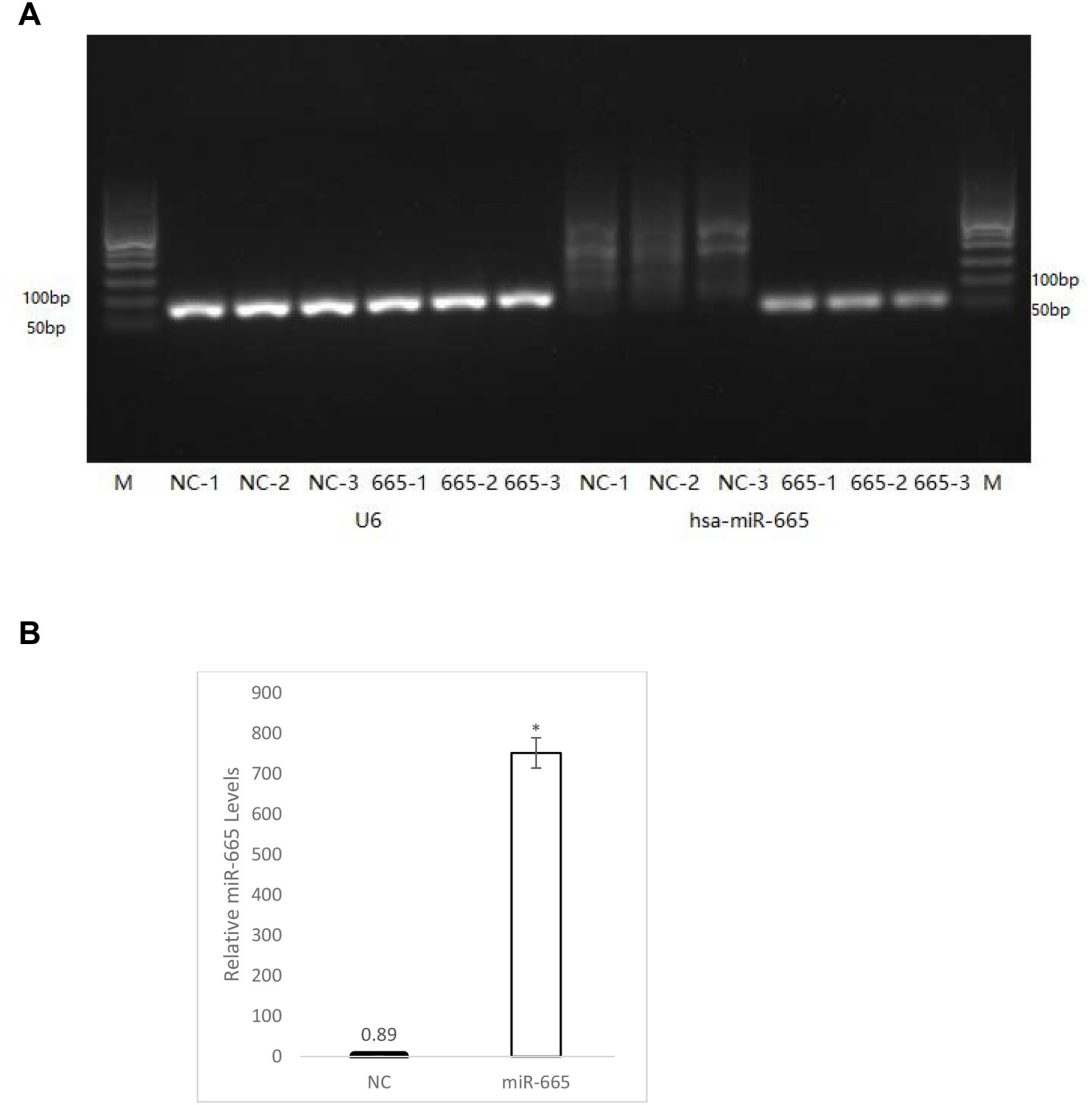
Quantitation of miR-665 in transfected cells miR-665 was quantitated via real-time qPCR normalized to the U6 gene from three biological replicates 48 h after transfection with negative control miRNA (cel-miR-67) or miR-665. From left, lane 1 and 14 (M), show DNA molecular weight ladder **(A)** Lanes 2–7 (NC-1–NC3 and 665-1–665-3) show the U6 gene. Lanes 8–10 (NC-1–NC3) show miR-665 levels from cells transfected with negative control miRNA. Lanes 11–13 (665-1–665-3) show miR-665 levels from cells transfected with miR-665. The miR-665 fold increase in miR-665-transfected cells was quantitated using the 2^-ΔΔCt^ method **(B)** miR665 levels are shown in cells transfected with negative control miRNA (black bar) and in miR-665-transfected cells (white). Error bars were calculated from the standard deviation from three biological replicates. ^*^P<0.54x10^-6^.

miR-665 levles were quantitated in neuroblastoma cells transfected with negative control miRNA and miR-665 using real time qPCR. Mouse neuroblastoma cells have very low levels of endogenous miR-665 (Figure 5A); however, miR-665 expression increased 848-fold in cells transfected with miR-665 mimic compared to cells transfected with the negative control miRNA, cel-miR-67 (Figure 5A–5B). miRNA levels reportedly increased by over 1000-fold in cells transfected with miR-200a [37]. Our results strongly indicate that miR-665 upregulation decreased MYC and HDAC8 expression, thus inhibiting proliferation and inducing apoptosis in mouse neuroblastoma cells.

### miR-665 directly targets HDAC8, c-MYC, and MYCN

**Figure 6:**
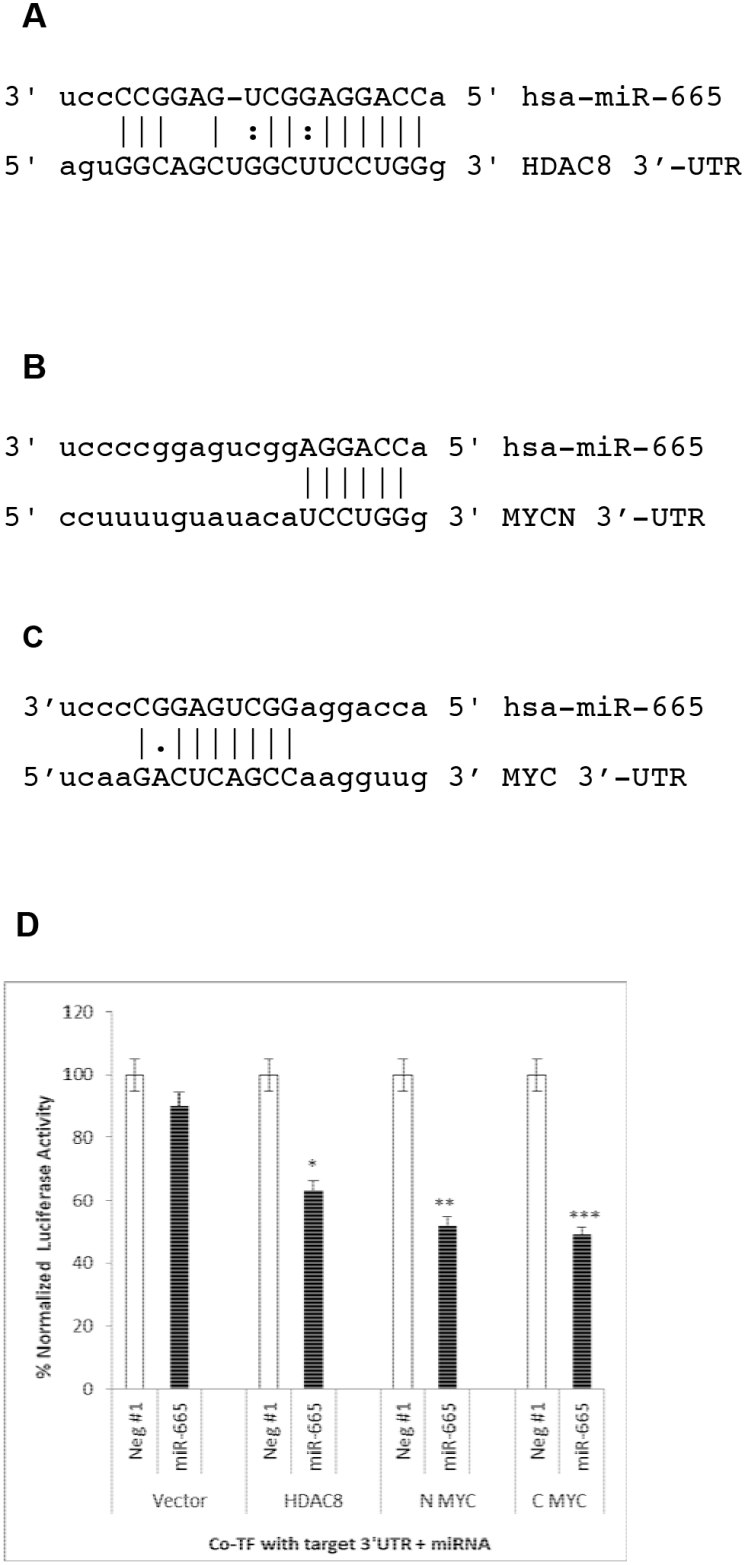
miR-665 directly targets the HDAC8, c-MYC, and MYCN 3’-UTRs miRanda (microRNA.org) predicted that hsa-miR-665 targets the 3’-UTRs of HDAC8 **(A)** and MYCN **(B)** miRanda and Targetscan did not identify the c-MYC 3’-UTR as an miR-665 target. Thus, complimentary sequences between miR-665 and the c-MYC 3’-UTR were compared using www.ebi.ac.uk **(C)** miR-665 targets were validated by co-transfecting luciferase expression plasmids with target 3’-UTRs and negative control miRNA or miR-665 into HepG2 cells **(D)** Empty vector with no target 3’-UTRs was used as a negative control. % luciferase activity is shown. SEM bars represent the standard deviation from two independent experiments with three biological replicates each. ^*^P<0.01, ^**^P<0.009, ^***^P<0.04.

We used the computational algorithm, miRanda (microRNA.org), to determine the mRNA targets of miR-665. The predicted targets for the human miRNA, hsa-miR-665, were the HDAC8 and MYCN 3’-UTRs (Figure 6A–6B). Although the miRanda and Targetscan algorithms did not indicate that miR-665 targeted the c-MYC 3’-UTR, we included this region in our analyses, as cMYC dysregulation is a hallmark of cancer. Furthermore, c-MYC is overexpressed in 30% of all human cancers and frequently predicts poor clinical outcomes [19, 20]. Our results indicated that miR-665 transfection downregulated the c-MYC protein by 40%. Therefore, we compared complementary sequences between miR-665 and c-MYC using a pair-wise sequence alignment website, www.ebi.ac.uk, and identified a potential binding site for miR-665 in the c-MYC 3’UTR (Figure 6C).

We tested whether or not miR-665 directly targeted HDAC8, c-MYC, and MYCN in HepG2 cells, because miR-665 does not inhibit HepG2 cell growth (unpublished data). Cells were co-transfected with a luciferase reporter expression plasmid containing a putative target gene 3’-UTR plus either the negative control miRNA or miR-665. Luciferase activity in cell extracts was measured 48 h later. Luciferase activity was decreased in cells co-transfected with miR-665 relative to those co-transfected with negative control miRNA.

Luciferase activity was decreased by 40%, 50%, and 51%, respectively, in cells co-transfected with miR-665 and plasmids containing the HDAC8 3’-UTR, the MYCN 3’-UTR, and the c-MYC 3’-UTR (Figure 6D). These results indicate that miR-665 directly targets the HDAC8, c- MYC, and MYCN mRNAs.

### Effects of siRNAs on neuroblastoma cells

**Figure 7:**
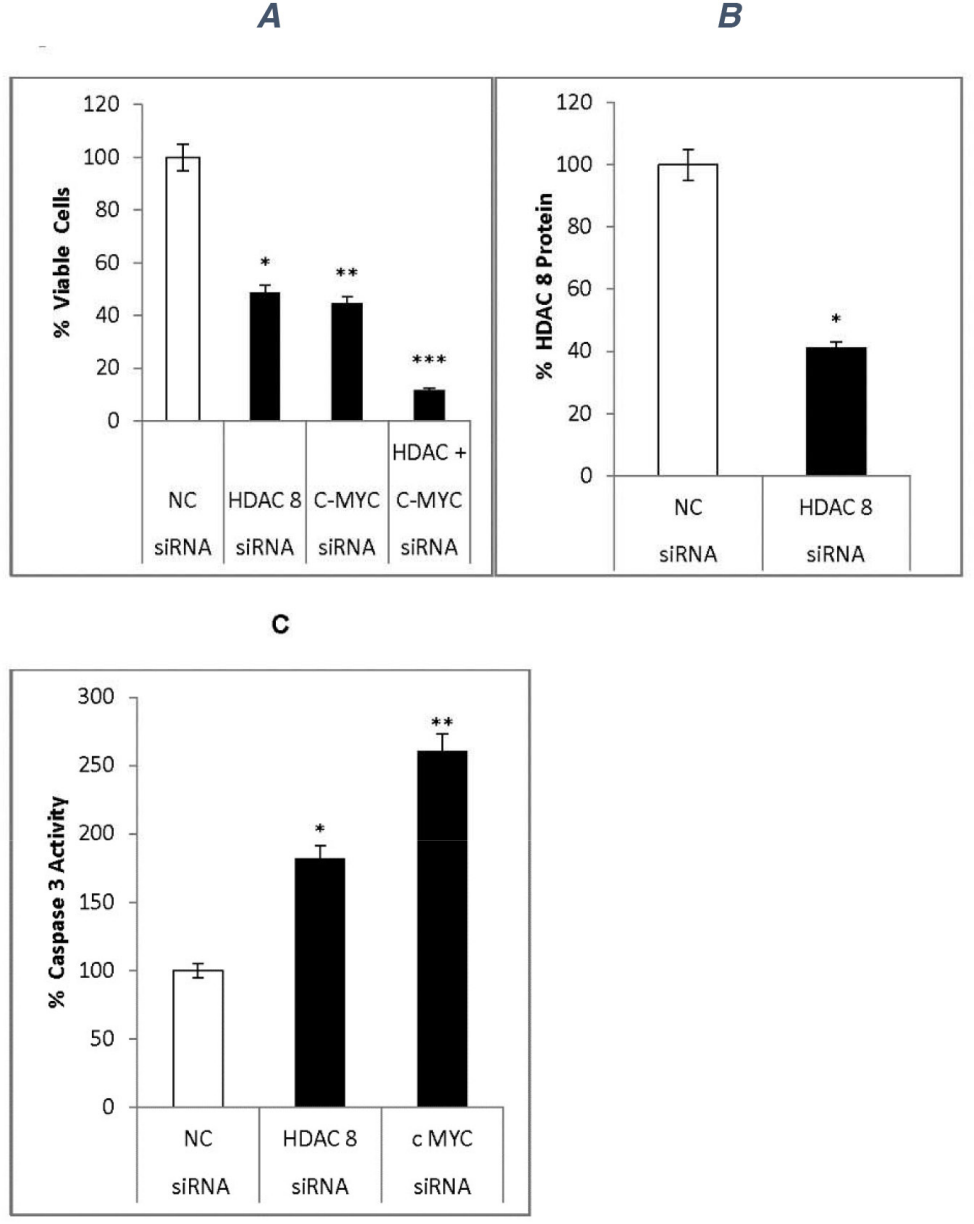
siRNA effects on neuroblastoma cells siRNA specific for HDAC8 (siRNA-HDAC8) or c-MYC (siRNA-c-MYC, a mixture of 4 siRNAs) were used to confirm the effects of miR665 in neuroblastoma cells. **(A)** siRNA effect on cell proliferation. Neuroblastoma cells were transfected with 50nM siRNA-HDAC8, 100nM siRNA-c-MYC, or both siRNAs together. Cell viability was measured via MTS assay. SEM bars represent the standard deviation from two independent experiments with three biological replicates each. ^*^P<0.005, ^**^P<0.001, ^***^P=6.8x10^-5^. **(B)** Cell extracts from negative control siRNA- or siRNA-HDAC8-treated cells were used to quantify HDAC8 levels via ELISA. HDAC8 was down regulated in HDAC8-siRNA-transfected cells ^*^P<0.04. **(C)** Caspase 3 activity was quantified in cell extracts via Casp-3 kit. Caspase 3 activity increased in siRNA-HDAC8- and siRNA-c-MYC-transfected cells. SEM bars represent the standard deviation from two independent experiments with two biological replicates each. ^*^P<0.01, ^**^P<0.004.

To verify miR-655 targets, we used siRNAs specific for HDAC8 (siRNA-HDAC8) and cMYC (siRNA-c-MYC). MYCN-specific siRNA was not used, because mouse neuroblastoma cells express low basal levels of MYCN.Neuroblastoma cells were transfected for 72 h with negative control siRNA, siRNA-HADC8, siRNA-c-MYC, or siRNA-HDAC8 + siRNA-c-MYC (Figure 7A). siRNA-HADC8 and siRNA-c-MYC inhibited cell proliferation by 42% and 55%, respectively. However, the combination of siRNA-HDAC8 + siRNA-c-MYC treatment inhibited proliferation by 86% (Figure 7A). The increased effectiveness of the siRNAs in combination appeared additive. These results indicate that HDAC8 and c-MYC are critical targets for reducing cell proliferation. Effective blockade of both targets is required to ensure a maximum inhibition of neuroblastoma cell proliferation.

### HDAC8 protein expression

HDAC8 protein levels were quantified via ELISA using extracts prepared from neuroblastoma cells transfected with either the negative control siRNA or siRNA-HDAC8.

HDAC8-siRNA inhibited HDAC8 expression by 40% (Figure 7B)

### Caspase 3 activity

Caspase 3 activity was measured in extracts prepared from cells transfected with various siRNAs. Compared to the negative control, siRNA-HDAC8 and siRNA-c-MYC increased caspase 3 activity by 1.8- and 2.5-fold, respectively (Figure 7C).

### Effects of miR-665 and siRNAs on histone acetylation

**Figure 8:**
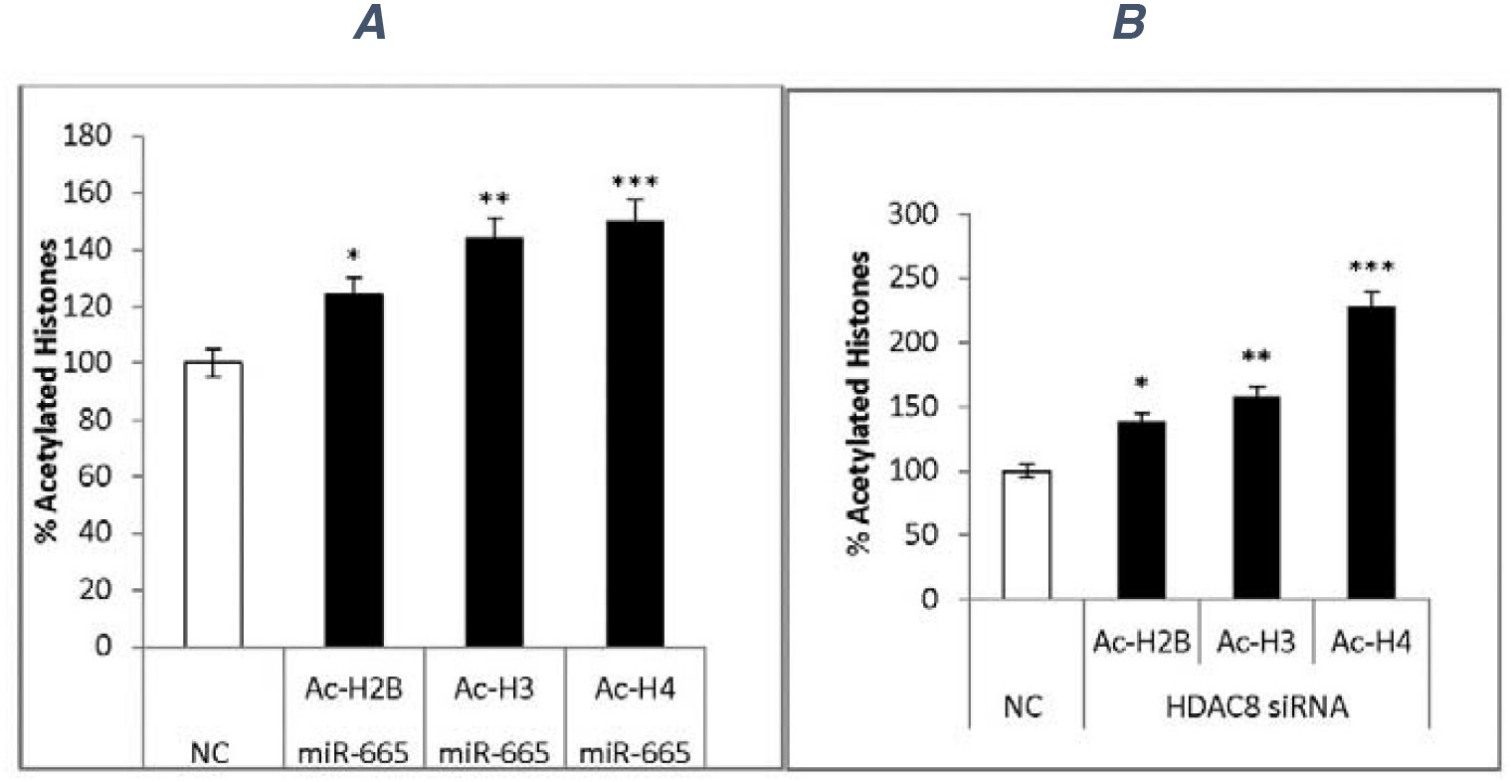
Histone acetylation Cell extracts from negative control miRNA-, miR-665-, negative control siRNA-, or siRNA-HDAC8 treated cells were used to quantitate histone acetylation via ELISA. Data represent standard deviations from two independent experiments. Negative control miRNA (white) and miR-665 transfection increased histone acetylation (black) **(A)** ^*^P<0.01, ^**^P<0.01, ^***^P<0.04. Negative control siRNA (white) and siRNA-HDAC8 increased histone acetylation (black) **(B)** ^*^P<0.008, ^**^P<0.04, ^***^P<0.001.

Our results indicated that miR-665 and siRNA-HDAC8 decreased total HDAC activity and specifically inhibited HDAC8 expression. miR-665 transfection increased acetylation of histones Ac-H2B (25%), Ac-H3 (40%), and Ac-H4 (50%) compared to negative controls (Figure 8A).

Likewise, siRNA-HDAC8 increased acetylation of histones Ac-H2B (38%), Ac-H3 (58%), and Ac-H4 (100%) (Figure 8B).

### miR-665 targets c-MYC and HDAC8

**Figure 9:**
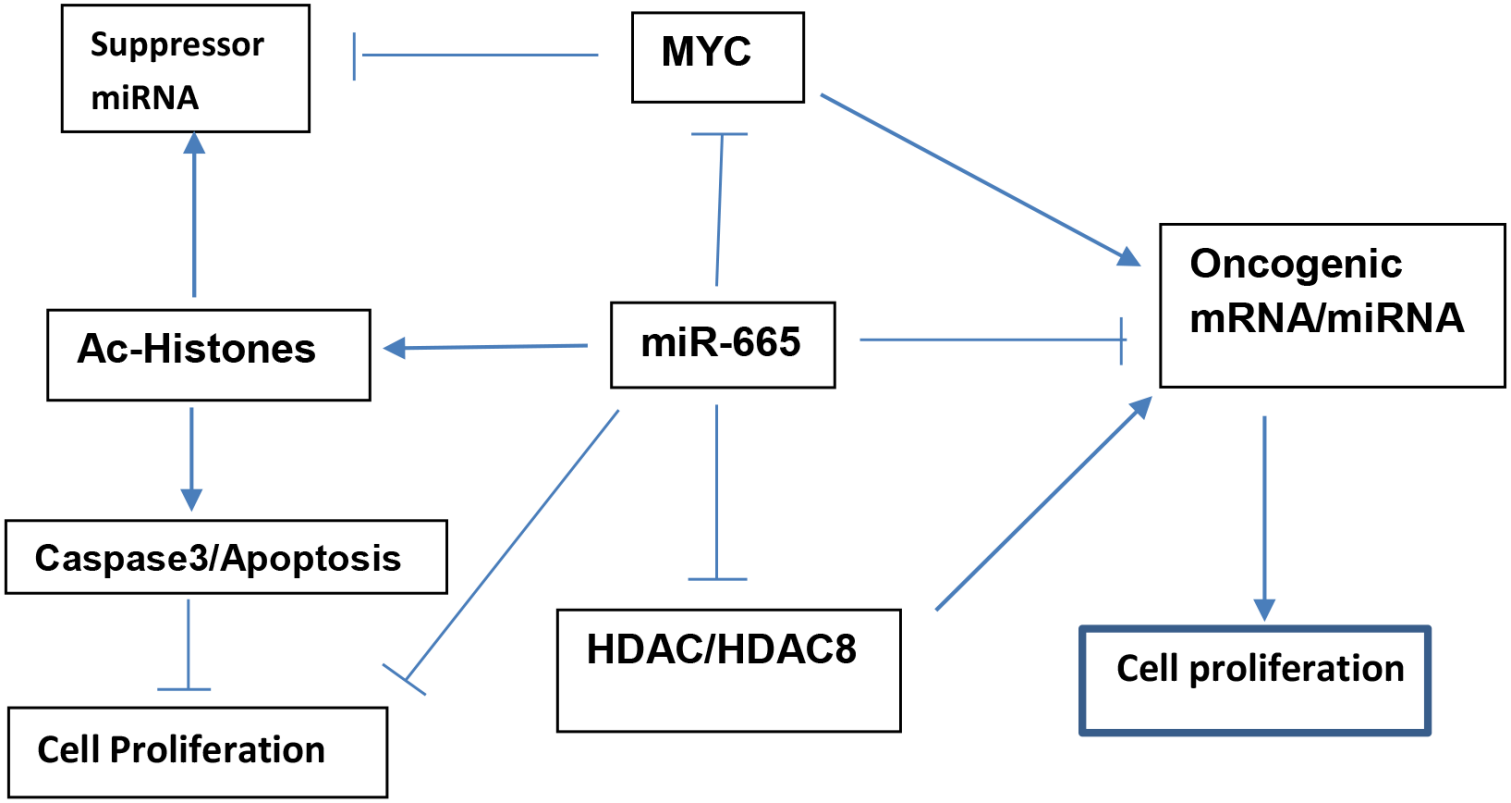
Proposed model illustrating how suppressor miR-665 targets c-MYC and HDAC8 to inhibit neuroblastoma cell proliferation and maintain cellular homeostasis

Taken together, our results indicate that miR-665 targets c-MYC and HDAC8, decreasing their expression, increasing histone acetylation, and modulating expression of cell proliferation related genes. We propose a model (Figure 9) illustrating suppressor miR-665 involvement in the inhibition of neuroblastoma cell proliferation.

## DISCUSSION

This study employed a mouse neuroblastoma cell model to study the role of miR-665 in neuroblastoma tumorigenesis. We found that miR-665 targets c-MYC and HDAC8 mRNAs to inhibit cell growth. This is the first report demonstrating that miR-665 is a suppressor miRNA targeting both c-MYC and HDAC8, which are involved in neuroblastoma tumorigenesis.

Our results indicate that Bt2cAMP upregulated suppressor miRNAs, and miR-665 transfection into neuroblastoma cells mimicked the effects of Bt2cAMP. Similarly, retinoic acid induced suppressor miR-34a, which targets MYCN, inhibits neuroblastoma cell growth and causes cell differentiation and apoptosis [15–18]. We found that miR-665 directly targets the cMYC and HDAC8 3’-UTRs, inhibiting their expression and reducing mouse neuroblastoma cell growth. miR-665 transfection led to accumulation of cells in G1 phase of the cell cycle, and reduced the number of cells in S phase. HDAC8 and c-MYC siRNAs activated caspase 3 and, consequently, caspase 3-dependent apoptosis. We speculate that miR-665 could potentially replace chemotherapeutic drugs as a novel anti-neuroblastoma therapy.

c-MYC and HDAC genes play critical roles in regulating the expression of several oncogenic and suppressor miRNAs. c-MYC is upregulated in many cancers, and is frequently involved in cancer pathogenesis [11, 20, 21]. MYC overexpression can upregulate oncogenic miRNAs, such as the miR-17-92 cluster directly activated in lymphoma [11], and can also repress several suppressor miRNAs [20]. HDACs regulate expression of genes involved in the cell cycle, apoptosis, and DNA synthesis [38]. HDAC8 plays a role in neuroblastoma pathogenesis, and HDAC8 overexpression was correlated with advanced neuroblastoma stage and metastasis [6]. siRNA-mediated HDAC8 knockdown inhibited proliferation of human lung, colon, cervical cancer, and neuroblastoma cell lines [6, 39]. Our results indicate a potential mechanism for these observations. Together, our findings indicate an inverse relationship between the levels of c-MYC and HDACs and suppressor miRNAs. When c-MYC and HDAC8 expression is high, as in tumor cells, then the levels of suppressor miRNAs will be lower; conversely, when c-MYC and HDAC8 expression is low, at basal levels, then suppressor miRNA levels will be higher.

Cellular homeostasis is maintained in part through equilibrium between oncogenic and suppressor miRNAs. Disruption of this equilibrium can cause cellular dysfunction, with oncogenic miRNA upregulation and suppressor miRNA downregulation leading to tumorigenesis [11, 40, 41]. However, addition of exogenous suppressor miRNAs to cancerous cells can reduce cell growth and promote apoptosis [10, 15, 17, 42–45]. Suppressor miR-122, miR-181, and miR-34a play critical roles in maintaining cellular homeostasis in liver and natural killer T-cells, and in colon cancer stem cells [46–48]. We speculate that suppressor miR-665 plays a critical role in neuroblastoma cell homeostasis. Our proposed model (Figure 9) illustrates how suppressor miR-665 targets c-MYC and HDAC8, and thus modulates cell proliferation to maintain cellular homeostasis.

Major advances have paved the way for safe, targeted miRNA- and siRNA-based therapies at the preclinical stage, and several miRNAs, anti-miRNAs, and siRNAs are in clinical trials as anti-cancer therapeutics [49]. Our laboratory is presently investigating the therapeutic effects of miR-665 and c-MYC- and HDAC8-specific siRNAs in transplanted mouse neuroblastoma tumors. In conclusion, this report is the first to identify miR-665 as a potent tumor suppressor that directly targets the 3’-UTRs of HDAC8, c-MYC, and MYCN, each of which is involved in neuroblastoma tumorigenesis. Our findings suggest that miR-665 might be a novel anti-neuroblastoma therapeutic.

## MATERIALS AND METHODS

### Reagents

Cell culture media, DMEM with high glucose (D6429), essential and non-essential amino acids (M5550, m7145), Bt2c AMP (D0627), the colorimetric Caspase 3 kit (Code CASP-3-C), and propidium iodide (P4170) were purchased from Sigma Aldrich, St. Louis. Fetal bovine serum (FBS) was purchased from Phenix Research Products, Candler, NC, USA. BD-Falcon tissue culture 96-well plates (353072) were purchased from BD-Biosciences. The RNA extraction miRNeasy kit (Cat No. 217084) was purchased from Qiagen, Germantown, MD, USA. The MTS Cell. Titer 96 Aqueous One Solution (Cat # G3580) cell proliferation assay was purchased from Promega Biotechnology, Madison,WI, USA. The HDAC Kit (#K331-100) was purchased from BioVision, Inc. Co rning. 96-well EIA/RIA plates (CLS3369) were used for ELISA. Antibodies for HDAC 8, H-145 (sc11405), C- MYC, C-19 (SC-786), acetylated Histones, Ac- H2B, Lys 5/12/15/20 (SC-8652), Ac-H3, lys9 (sc-8655), Ac-H4, lys16 (sc-8662), and siRNA for c-MYC (pool of 4 different siRNA duplexes, sc-29227) were purchased from Santa Cruz Biotechnology, Dallas, TX, USA. Negative control #2 siRNA (#4390846), siRNA-HDAC 8 (S88696) and Lipofectamine RNAi Max (#13778075) were purchased from Life Technologies/Ambion/ Invitrogen. Negative control miRNA Cel-miR-67 (#CN-001000) sequences based on C. elegans miRNA, mimic hsa-miR-665 (#C-301246-01), and transfection reagent Dharmafect Duo (#T2010-01) were purchased from Dharmacon. Luciferase expression plasmids with the 3’-UTR for HDAC8 (#S804229), C-MYC (#S804638), MYCN (Product No S807230), or empty vector without 3’-UTR (#S890005), and the LightSwitch luciferase assay kit (#32031, LS010) were purchased from Active Motif, CA, USA.

### Cells and cell culture

Mouse neuroblastoma cholinergic clonal cells (S20) were obtained from Dr. Marshall Nirenberg of The US National Institutes of Health (NIH). Cells were grown in monolayers in DMEM supplemented with essential and nonessential amino acids, penicillin/streptomycin, and 10% FBS at 37°C with 5% CO_2_ and humidity.

### Bt2cAMP treatment

Neuroblastoma cells were plated in 96-well plates at 12x10^3^ cells per well and treated with 1mM Bt2cAMP. After 48–72 h, cell viability was measured colorimetrically using the MTS Cell Titer 96 Aqueous One Solution. Samples were incubated at 37°C for 3–4 h and samples were read at 490 nm in a plate reader according to the manufacturer’s instructions.

For cell cycle analysis, 1x10^6^ cells were plated in T25 flasks and treated with 1mM Bt2cAMP. After 48 h, cells were trypsinized, treated with 75% ethanol and 100ug/ml RNAse A, and then stained with propodium iodide (PI). Untreated and Bt2cAMP-treated (20,000 cells/sample) were analyzed for cell cycle distribution via flow cytometry at the Core lab of Children’s Cancer Center Hospital, Houston, TX, USA.

### RNA extraction and miRNA differential expression

Untreated (control) and 1mM Bt2cAMP-treated neuroblastoma cells were grown in T75 flasks in three biological replicates per treatment. After 72 h, total RNA was extracted using Qiagen’s miRNeasy RNA extraction kit. miRNA expression was analyzed by Exiqon, Copenhagen, Denmark. RNA samples were labeled with fluorescent Hy3 and Hy5 using the miRCURY LNA Array power labeling kit. Labeled samples and labeled reference RNA samples were mixed pair-wise and hybridized to the miRCURY LNA array v.11.0 (Exiqon), which contained captured probes targeting all human, mouse, or rat miRNAs registered in the Sanger Institute miRBASE v.14.0.

### Transfections

The effects of miR-665 or siRNA on cell proliferation were determined using reverse transfection. First, 100nM negative control miRNA, miR-665, negative control siRNA, C-MYC siRNA, or HDAC8 siRNA was mixed with Lipofectamine RNAimax. This mixture was added to 12x10^3^ cells, which were then plated in 96-well plates. After 48–72 h, cell viability was measured using MTS Cell Titer 96 Aqueous One Solution and incubated at 37°C for 3–4 h. Samples were read at 490nm according to manufacturer’s instructions.

miRNA effects on the cell cycle were assessed using reverse transfection of cells with 100nM negative control miRNA or miR-665 mimic plus Lipofectamine RNAimax. The transfection mixture was added to 1x10^6^ cells, which were then plated in a T25 flask. After 48 h, cells were trypsinized, treated with 75% ethanol and 100ug/ml RNAse A, and then stained with PI. For cell cycle analysis, 20,000 cells/sample were analyzed via flow cytometry in the Core lab of Children’s Cancer Center Hospital, Houston, TX.

### Whole cell extracts

Cell extracts were prepared from untreated, 1mM Bt2cAMP-treated (72 h treatment), and miRNA-transfected cells for target assays. miRNA-transfected cells were reverse transfected with 100nM negative control miRNA, miR-665, negative control siRNA, c-MYC siRNA, or HDAC8 siRNA plus Lipofectamine RNAimax. Transfected cells were plated inT25 flasks. After 48–72 h, cell extracts were prepared in assay buffer as described by Khandelia, *et al.* [50]. Assay buffer consisted of 20mM Tris-HCL pH 7.5, 150mM NaCl, 5mM EDTA, 10% glycerol, 1% Nonidet P40, and protease inhibitor cocktail from Sigma (P8340). Protein concentrations were determined using Pierce’s BCA Assay as per the manufacturer’s instructions.

Bt2cAMP and miR-665 inhibit cell growth compared to untreated cells and cells treated with negative control miRNA. Assays were normalized using equal concentrations of protein (50–100 ug) from untreated, negative control miRNA-, Bt2cAMP-, and miR-665-treated cells in assessing total HDAC and Caspase 3 activity, and HDAC8 and c-MYC levels via ELISA.

### Quantitation of miR-665 in transfected cells

Mouse neuroblastoma cells were transfected with 100nM miR-665 mimic and negative control cel-miR-67. 48 h post-transfection, total RNA was extracted from three biological replicates per treatment using the Qiagen RNEasy mini kit. miR-665 was quantitated via realtime qPCR by Arraystar, Inc (Rockville, MD, USA).

Real-time PCR was performed for each RNA sample to quantify miR-665 and the housekeeping gene, U6. According to the standard curve, mRNA concentrations in each sample are determined directly using Rotor-Gene Real-Time Analysis software v.6.0 and the 2^∆∆Ct^ method.

### Total HDAC activity

Total HDAC activity was measured in 50–75ug of protein from cell extracts prepared from untreated, Bt2cAMP-treated, or negative control miRNA- or miR-665-transfected cells using the Biovision kit (#K331-100). Acetylated HDAC substrate and other reagents were added according to the manufacturer’s instructions and the final deacetylated product was read at 405 nm in a plate reader.

### HDAC8 and c-MYC protein quantitation via ELISA

HDAC8 and c-MYC proteins were quantitated using cell extracts prepared from untreated or 1mM Bt2cAMP-treated cells, or negative control miRNA- or miR-665-transfected cells via ELISA. 100ug protein per sample was mixed with 0.02M carbonate coating buffer (pH 9.5) and added to 96-well BD-Falcon ELISA plates. Samples were incubated at 4°C for 15 h. Wells were blocked with 10% FBS in PBS, treated with antibodies (diluted 1:30) specific for HDAC8 (SC11405) or c-MYC (SC-798), and incubated at 37°C for 2 h. Samples were washed with PBS + 0.05% Tween, treated with goat anti-rabbit IgG-HRP secondary antibody (diluted 1:500), and incubated at 37°C for 1 h. Wells were washed and treated with substrate TMB and incubated at room temperature for 30 min, and then the reaction was stopped with 2N H_2_SO_4_. Samples were read at 450 nm in a plate reader.

### Caspase 3 activity

Caspase 3 activity was measured in 50ug protein from untreated, Bt2cAMP-treated, or miR-665-transfected cells using Sigma Aldrich’s colorimeter kit (Code CASP3-C). 50ug protein was mixed with the peptide substrate, Ac-DEVD-pNA (p-nitroanilide), in the presence of 10mM DTT. Caspase 3 hydrolyzes the substrate, releasing p-nitroaniline, which is read at 405 nm. The specificity of caspase 3 activity was determined in the presence of the inhibitor, Ac-DEVD- CHO.

### Target validation using luciferase expression plasmids

HepG2 cells were used for miR-665 target validation, because miR-665 does not inhibit the growth of these cells. When mouse neuroblastoma cells were used for target validation, the negative control luciferase vector plasmid without any target 3’-UTR showed a 50% decrease in luciferase activity when co-transfected with miR-665 compared to negative control miRNA. This decrease in luciferase activity was non-specific and was caused by cell growth inhibition due to miR-665 transfection.

To validate miR-665 targets, HepG2 cells were grown for 24 h in a 96-well plate. Cells were then co-transfected with 100ng luciferase expression plasmids containing the 3’-UTR for HDAC8, c-MYC, or MYCN, or the empty vector without any target 3’UTR, plus 100nM negative control miRNA or miR-665 with Dharmafect Duo transfection agent. After 48 h of cotransfection, luciferase activity was measured using the Active Motifs LightSwitch luciferase assay kit. Luminescence was read on a Molecular Devices Soft Max Pro5 luminometer.

### Histone acetylation

Histone acetylation was quantified via ELISA in cell extracts prepared from cells transfected with negative control miRNA, miR-665, negative control siRNA, or HDAC8 siRNA. 100ug protein was mixed with 0.02M carbonate coating buffer (pH 9.5), added to 96-well BDFalcon ELISA plates, and incubated at 4°C for 15 h. Wells were blocked with 10% FBS in PBS, treated with acetylated antibodies (diluted 1:30) for Ac-H2B (Lys 5/12/15/20), Ac-H3 (lys9), or Ac-H4 (lys16), and incubated at 37°C for 2 h. Samples were washed with PBS + 0.05% Tween, treated with an appropriate HRP-conjugated secondary antibody (diluted 1:500), and incubated at 37°C for 1 h. Wells were washed and treated with substrate TMB and incubated at room temperature for 30 min, and then the reaction was stopped using 2N H_2_SO_4_. Samples were read at 450 nm in a plate reader.

### Statistical analysis

Error bars represent standard error of the mean (SEM) from 2–3 biological replicates from 3–5 independent experiments. P-values were calculated using T.Test (2 tailed, 3 samples, unequal variance) and p<0.05 was considered statistically significant.
